# Predictive Value of Auricular Diagnosis on Coronary Heart Disease

**DOI:** 10.1155/2012/706249

**Published:** 2012-12-24

**Authors:** Lorna Kwai-Ping Suen, Yuk-kong Lau, Hok-cheung Ma, Kam-wai Lai, Eleanor Holroyd

**Affiliations:** ^1^School of Nursing, The Hong Kong Polytechnic University, Hung Hom, Hong Kong; ^2^Ruttonjee Hospital and Tang Shiu Kin Hospital, Hong Kong; ^3^Caritas Medical Centre, Hong Kong; ^4^Grantham Hospital, Hong Kong; ^5^Division of Nursing and Midwifery, School of Health Sciences, RMIT University, Australia

## Abstract

The ear has a reflexive property; therefore, various physical attributes may appear on the auricle when disorders of the internal organs or other parts of the body exist. Auricular diagnostics is an objective, painless, and noninvasive method that provides rapid access to information. Thus, the association between auricular signals and coronary heart disease (CHD) should be further investigated. A case control study was conducted to determine the predictive value of auricular signals on 100 cases of CHD (CHD+ve = 50; CHD−ve = 50) via visual inspection, electrical skin resistance measurement, and tenderness testing. The results showed that the presence of an ear lobe crease (ELC) was significantly associated with coronary heart disease. The “heart” zone of the CHD+ve group significantly exhibited higher conductivity on both ears than that of the controls. The CHD+ve group experienced significant tenderness in the “heart” region compared with those in the CHD−ve group in both acute and chronic conditions. Further studies that take into consideration the impact of age, race, and earlobe shape on ELC prevalence in a larger sample should be done.

## 1. Introduction

The ear was first mentioned in the earliest Chinese medical book, *Yellow Emperor's Classics of Internal Medicine*, which was published more than 2,000 years ago. The book states that the ear is related to all parts of the human body and internal organs and that all meridians converge at the ear. The Chinese Association of Acupuncture and Moxibustion was tasked by the World Health Organization to standardize acupoint locations to have a common language in the study and exchange of ideas. In May 1993, the “Chinese Standard Ear-Acupoint the Chinese Chart,” which consists of 91 acupoints, was established [1].

The ear has a reflexive property; therefore, various physical attributes may appear on the auricle when body disorders exist. Such attributes include variations in shape, color, size, and sensation; appearance of papules, creases, and edema; and increased tenderness or decreased electrical conductivity [2, 3]. The association between the presence of an ear lobe crease (ELC) and coronary heart disease (CHD) was first reported by Frank [4] who identified diagonal creases on the ear lobes that run either unilaterally or bilaterally from the lower probe of the external auditory meatus diagonally backwards to the edge of the lobe [5]. Subsequently, several studies have demonstrated similar associations, either in patients with CHD [6–8] or in forensic autopsy cases [9, 10].

Other auricular signs associated with body functions also exist. For example, a published paper has reported that the presence of ELC has a high CHD predictive value [11]. Meanwhile, another study has reported that abnormal hair growth on the ear is a sign of hormonal changes that accompany the decline of Kidney *Qi*, which occurs with aging [2]. From the TCM perspective, the presence of ear hair is associated with blood lipids or other auricular signals [12]. Other possible reactive signs that appear on the ears in patients with CHD include tenderness, discoloration, and marks or edema ripples that appear after pressing the “Heart” region of the ear [13]. In addition, according to the auricular diagnosis system, the areas of the auricle where increased electrical conductivity and heightened tenderness appear upon touching correspond to specific areas of the body where some pathological conditions exist [14].

Despite the decline in age-standardized death rates of CHD patients in the last two decades, deaths due to CHD accounts for 70% of the mortality rate of heart disease [15]. Therefore, the early diagnosis of CHD through a non-invasive and effective approach is necessary. Auricular diagnosis is an objective, painless, and non-invasive method that provides rapid access to information. Thus, the association between auricular signals and CHD should be further investigated. Such an investigation within the Hong Kong context would help extrapolate the findings to the Chinese population for purposes of cardiac risk assessment. Therefore, a pilot case control study was conducted to examine the association of these auricular signals with CHD.

## 2. Aim and Hypotheses

**Figure 1:**
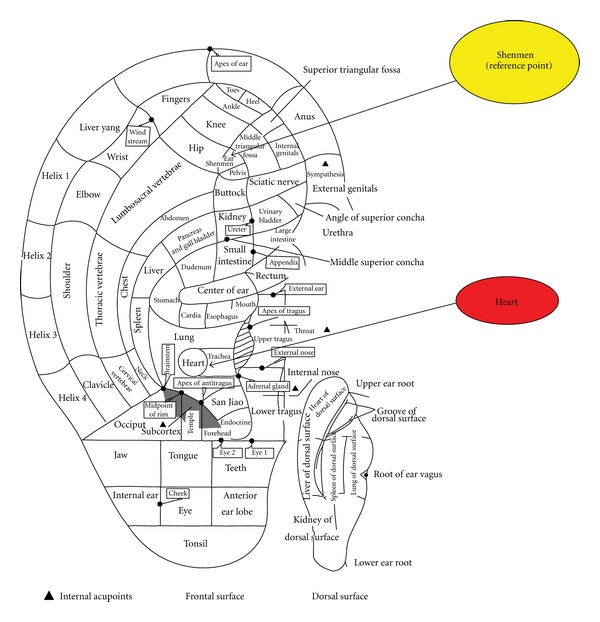
The “Heart” and the “Shenmen” (reference) points on the auricle.

This study aims to investigate the predictive value of auricular diagnosis on CHD. The hypotheses of this study are as follows: (1) visual inspection results indicated that patients with CHD exhibit a higher occurrence of ELC, appearance of edema ripples in the “Heart” region after pressing, and hair growth on the ears compared with community control group; (2) patients with CHD have lower electrical skin resistance on the “Heart” region of the ears than the community control group; (3) patients with CHD have significant tenderness on the “Heart” region of the ears than the community control group; (4) the degree of stenosis of the coronary arteries and the number of major epicardial arteries involved in CHD patients are associated with the presence of ELC; (5) a significant association exists between lipid profile (cholesterol and triglycerides) and the presence of hair growth or other auricular signals. The location of the “Heart” region in the Chinese Standard Ear-Acupoint Chart is displayed in Figure 1.

## 3. Methods

### 3.1. Settings and Participants

This pilot study is a case-control study. Potential subjects were recruited from a regional hospital in Hong Kong (CHD+ve) and from the community (CHD−ve). Fifty subjects from each group were recruited through convenience sampling. The subjects from the CHD+ve group were recruited from the cardiac unit of the target hospital. Half of the 50 subjects suffered from acute episodes (*n* = 25), that is, newly diagnosed of having CHD within three months, whereas the rest were chronic CHD cases (*n* = 25). All CHD+ve cases were compared with the community subjects without CHD (CHD−ve group), who did not have any previous medical history of CHD or any experience of having cardiac-related symptoms including palpitation, chest pain, and/or dyspnoea. The control group was recruited from hospital staff, relatives, or friends of CHD patients. The participants in the case and control groups were matched by age and gender. Only the subjects who were aged 18 years or above were recruited, and patients with aural injury or infections were excluded from the study.

### 3.2. Data Collection

A simple health assessment was conducted (e.g., blood pressure reading, body mass index (BMI), medical and family history, fasting cholesterol, triglycerides, and glucose) on the participants. Auricular signals that appear on patients with CHD (CHD+ve group, *n* = 50) were compared with patients without evidence of CHD (CHD−ve group, *n* = 50). Ear diagnosis was conducted in three ways: through inspection, palpation, and electrical detection. The observer was blinded to the grouping of the participants. Special auricular signals that were associated with coronary risks were observed and recorded on both ears.

Comprehensive records include an examination of tenderness (using digital force gauge), appearance of edema ripples in “Heart” region after pressing, presence of ELC, presence of ear hairs, and electrical resistance detected on the “Heart” region.

The auricles were assessed based on the following procedures.

**(a) Visual Inspection**

Both auricles were observed for discoloration, appearance of edema ripples in “Heart” region after pressing, presence of ear hair, and ELC. ELC was graded according to the system modified from Patel et al. [10]: grade 0: no crease at all; grade 1: any crease greater than 0% but less than or equal to 50%; grade 2: greater than 50% but less than 100% across the lobe; grade 3: a complete crease across the lobe that is superficial but not deep; and grade 4: deep and prominent crease across the whole lobe.

**(b) Electrical Skin Resistance Measurement**

An individual threshold was set for each subject before assessing the ear acupoints. The threshold was obtained by placing the acupoint detector on the “Shenmen” point and increasing the detection sensitivity until the sound, lights, or visual meter on the equipment indicates a high electrical conductance. The sensitivity was then slightly reduced until the “Shenmen” point was only barely picked up [1, 3]. An electrical acupoint detector (Pointer Plus) was used to measure the auricular electrical resistance in the “Heart” region. The detector would emit a beeping sound, and its green light would flash upon detecting the presence of electrical conductivity in the “Heart” region (i.e., the “Heart” zone has lower electrical skin resistance than the “Shenmen” reference point).

**(c) Tenderness Testing**

A pressure algometer (force gauge) with a unit range of 0 g to 500 g was used to apply force in the “Heart” zone using the “Shenmen” point as reference. The observed value (g) was recorded each time the subject starts feeling pain when the pointer of the instrument was applied on the acupoints during testing.

**(d) Other Parameters**

A checklist was used to facilitate data collection. The data on coronary risk factors was collected. Increasing age, male gender, and heredity are the risk factors that cannot be changed; however, tobacco smoking, high blood cholesterol level, hypertension, obesity, and diabetes mellitus are modifiable risk factors.

The findings of previous coronary angiography (within six months) for the CHD+ve group were retrieved to assess the severity of coronary artery stenosis (%) and the number of major epicardial arteries involved. The patients were divided into two groups: those with significant coronary atherosclerosis (≥50% stenosis in at least one of the major coronary arteries) and those with negligible or low degree stenosis (<50%), according to the criteria set by Kaukola et al. [16]. The number of vessels with blockage (multivessel versus 1-vessel disease) was measured using the standard set by Tranchesi et al. [6].

## 4. Validity and Reliability

The inter-observer agreement of observations by the Principal Investigator and the Research Assistant (RA) was recorded for ten cases to assess the reliability of the auricular examinations. Digital photos of the auricles were obtained to examine the inter-observer agreement of the ELC grading using Kappa's statistic, thereby increasing the validity of the findings [17]. Both the researcher (L.K-P. Suen) and the RA were blinded to the patient groupings during the examination of the photos for the remaining samples. A consensus was sought after discussion when the perceived ELC percentage between the two raters was greater than 10%. The raters were blinded to the grouping of subjects to avoid observer bias. 

## 5. Data Analyses

Sensitivity, specificity, positive predictive value (PPV), and negative predictive value (NPV) with 95% confidence intervals (Cls), as well as odds ratios of auricular signals (presence or absence of ELC, discoloration, hair growth, marks, edema, tenderness, and electrical resistance) that predict the CHD were computed. ELC grading was converted to percentage (from 0% to 100%), and a receiver operator characteristics (ROC) curve was plotted to produce a table of sensitivity and specificity for different cutoff scores. Kappa's statistic was computed to determine agreements on CHD status (+ve versus −ve) and those classified by different cutoff points of ELC%. Chi-square test was used to determine the association between the auricular signals and CHD (+ve versus −ve) and between auricular signals and the degree of coronary stenosis (≥50% versus <50%, 1-vessel versus multivessel disease). The Student's *t*-test was used to compare the mean scores of the continuous variables between the case and control groups, as well as between CHD patients with acute episodes and those with chronic conditions, when appropriate. These variables were namely the body mass index, lipid profile, ELC%, and electrical resistance readings of individual acupoints. A multivariate logistic regression analysis was conducted to examine the association of the cardiovascular status of the subjects with the auricular signals after controlling for potential confounders. The auricular signals included the presence or absence of ELC, hair growth, edema ripples, tenderness, and the conductivity of the “Heart” region. Analyses were conducted using SPSS Statistics 19. A value of *P* < 0.05 was considered statistically significant.

## 6. Ethical Considerations

Ethical approval from the hospital and the university was sought. Written informed consent was obtained from every eligible person who agreed to participate. The purpose and procedures of the study were explained verbally and in writing to the participants. Participation in the study was on a voluntary basis, and all participants were assured that they have the right to refuse or withdraw from the study at any time. Personal information and data remained confidential and anonymous.

## 7. Results

### 7.1. Demographic Characteristics

A total of 100 participants were recruited, among which 50 were from a regional hospital in Hong Kong (25 acute cases and 25 chronic cases) and 50 from the community (CHD−ve as controls). The participants in the case and control groups were matched by age and gender. The mean age of the participants was 65.32 years (S.D. = 14.12), with 54 males and 46 females. The mean ages of the participants for the acute and chronic cases were 64.92 years (S.D. = 14.55) and 65.56 years (S.D. = 14.76), respectively, with no significant gender distribution between groups. No significant differences were observed in the BMI, presence of comorbid illness, and the family history of myocardial infarction between the groups. However, participants in the CHD+ve group have more ex-smokers/smokers (*P* > 0.05) with slightly lower mean arterial pressure (MAP; *P* > 0.05) than those in the CHD-ve group. Therefore, these two variables were adjusted for the subsequent multivariate analyses (Table 1).

### 7.2. Auricular Signals

The auricular signals that appear on patients with CHD (CHD+ve) were compared with those of patients without evidence of CHD (CHD−ve).

**(1) Visual Inspection**

**Figure 2:**
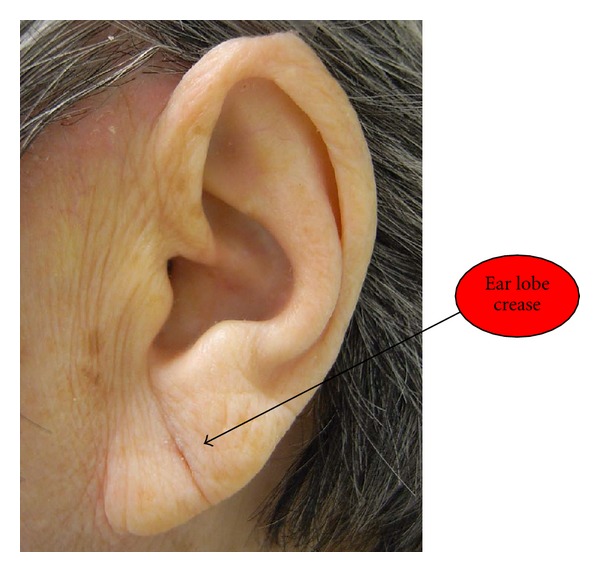
A complete ear lobe crease on the left-side of ear of a female participant having an acute CHD condition.

The presence of ELC, which is the primary auricular signal for CHD prediction in this study, was significantly associated with CHD. More participants in the CHD+ve group showed the presence of ELC compared with those in the CHD−ve group (*P* < 0.05; Figure 2). An average of 47.5% versus 33.0% of crease across the ear lobe was noted on the left ear (*P* < 0.05), whereas 43.1% versus 24.0% was noted on the right ear (*P* < 0.05) in the CHD+ve and CHD−ve groups, respectively. In addition, more participants in the CHD+ve group exhibited hair growth on ears than those in the CHD−ve group, especially on the right ear (*P* < 0.01). The results from the stratified analyses indicated that the average percentage of ELC across the ear lobe among cases with acute condition was significantly different from the control group, whereas the chronic cases were found to have significant hair growth on right ear.

A significant relationship between the presence of ELC on the right and left ears was observed in all cases (*n* = 100, *r* = 0.711, *P* < 0.001). Bilateral ELCs were present in 45% (*n* = 45) of the participants, whereas 13% showed one-sided ELC. The ELC sign on the right (*r* = 0.546, *P* < 0.001) and left ears (*r* = 0.441, *P* < 0.001) was associated with increasing age, but was independently associated with gender. Edema ripples in the “Heart” region after pressing appeared in most participants of the CHD+ve group (*P* < 0.05). Among these CHD+ve cases, the most commonly involved artery with blockage was the left anterior descending (LAD, 75%), followed by the right coronary artery (RCA, 55.3%), left circumflex (LCX, 44.7%), posterior descending artery (PDA, 16.2%), and posterior lateral branch (PLB, 2.6%). Among these arteries, the degree of RCA stenosis was significantly associated with the presence of ELC on either the right (*P* < 0.001) or left ear (*P* < 0.05). Moreover, the number of major epicardial arteries involved was also associated with the presence of ELC (*P* < 0.01, trend analysis = 0.02) and edema around the “Heart” zone (*P* < 0.05) on the right ear.

Among these auricular signs, ELC sensitivity was 61.0%, specificity was 58.0%, and PPV was 59.3%. An ROC curve was generated to produce the best cut-point analysis. The use of 35% ELC across the ear lobe was the most desirable cutoff value for predicting CHD status, which was supported by a fair agreement of Kappa's statistic in either the right (Kappa = 0.28, *P* < 0.05, AUC = 0.64, sensitivity = 0.80, specificity = 0.48) or left ear (Kappa = 0.36, *P* < 0.01, AUC = 0.68, sensitivity = 0.96, specificity = 0.40).

**(2) Electrical Skin Resistance Measurement**

The “Heart” zone of the CHD+ve cases had a significantly higher conductivity on both ears (i.e., less electrical skin resistance) compared with the control group (*P* < 0.01). The results from the stratified analyses revealed a significant change in the conductivity of the “Heart” zone in chronic CHD+ve cases (*P* < 0.01), but not in acute cases. The conductivity of the “Heart” zone exhibited a sensitivity of 49.0%, a specificity of 80.0%, and a PPV of 71.7%. However, no association was observed between the electrical conductivity of the “Heart zone” and the degree of stenosis of the coronary arteries and the number of major epicardial arteries involved.

**(3) Tenderness Testing**

The participants in the CHD+ve group experienced significant tenderness in the “Heart” region in both ears (*P* < 0.001) compared with those in the CHD−ve group. A significant relationship was noted between the average tenderness (%) on both ears (*r* = 0.707, *P* < 0.001). The results from the stratified analyses indicated that these two signals were found significant in cases with either acute (*P* < 0.01) or chronic condition (*P* < 0.01). The result from the tenderness testing of the “Heart” zone showed a sensitivity of 92.0%, a specificity 75.0%, and a PPV of 78.6%. No association was observed between tenderness of the “Heart” zone and the degree of stenosis of the coronary arteries and the number of major epicardial arteries involved.

### 7.3. Multivariate Analyses

Multivariate logistic regression analysis was conducted to examine the association of the cardiovascular status of the subjects with a number of auricular signals after controlling for the significant risk factors (smoking status and MAP). Age and gender were not included in the model because the cases and controls were matched according to these attributes. Backward logistic regression was used, and auricular signals that demonstrated statistical significance (*P* < 0.05) were retained in the final model (Table 2). The overall multivariate model was significant (*P* < 0.001) and correctly classified in 88.0% of the subjects. The result of the Hosmer and Lemeshow test indicated that the model effectively fits with the data (*P* = 0.081). In this final model, each 1% increase in the ELC occurrence on the left side of the ear will increase the risk of CHD by 1.017 times (adjusted OR = 1.017, 95% CI = 1.000–1.035). The presence of tenderness (adjusted OR = 49.99, 95% CI = 11.81–211.58) and conductivity (adjusted OR = 5.67, 95% CI = 1.20–26.69) in the “Heart” region of the left ear was likewise significantly associated with the CHD status after the adjustment of the smoking status and MAP readings. However, when the relationships among the three auricular signals were examined, all their associations on the left and right ears as well as in the acute and chronic cases were not significant.

## 8. Discussion

This study adopted a systematic and scientific approach and used visual inspection, electrical skin resistance measurement, and tenderness testing to investigate the auricular signals and their relationships with CHD. Coronary angiographic is a reliable objective measure and has long been considered as the “gold standard” for CHD diagnosis [16]. This examination was adopted in this study, where the association between angiography findings and auricular signals was observed. The results of this study suggest that the presence of ELC, high electrical conductivity, and tenderness of the “heart” region were associated with the presence of CHD. These findings can advance our knowledge on the integrated approach in combining Chinese and Western models of care for diagnosing patients with underlying CHD. 

### 8.1. Visual Inspection

More participants in the CHD+ve group exhibited the presence of ELC compared with those in the CHD−ve group. This result supports the findings of a number of previous studies [11, 18–21], where the presence of ELC is positively associated with CHD. The relationship between diagonal ELC and premature cardiovascular disease was first described by Frank in 1973 [4]. The French physician P. Nogier also put forward a hypothesis that the human body can be projected into the auricle in the same way as it is projected into the brain cortex [22]. Although negative claims have been made regarding the association between ELC and CHD, most of these claims were made in the 90s [23, 24]. Several positive claims that support the independent association of ELC with the prevalence and severity of CHD have been widely reported in more recent years [25–27].

Although the occurrence of ELC may increase with age [28–30], our results showed an increased ELC frequency among CHD patients that was independent of age because both case and control groups were matched by age and gender. ELC sensitivity was 61.0%, specificity was 58.0%, and PPV was 59.3% for predicting the CHD status. However, wide variations exist in the report of these values from previous literature, which ranged from 51.0% to 65.0% in sensitivity, 72.0% to 94.0% in specificity, and 42.0% to 91.1% in PPV [6, 31, 32]. The low specificity (58.0%) of ELC in this study as compared with the literature could be attributed to the different scoring systems that were adopted in the different studies [10, 31, 32]. The present study adopted an ELC grading system that was modified from Patel et al. [10]. A significant relationship between the presence of ELC on the right and left ears was noted. ELC usually appeared bilaterally (45.0%) rather than just on one side of the ear (13.0%).

Several researchers believed that creasing is related to earlobe shape, variation in age of creasing onset according to race, and the variation in the frequencies of occurrence of different earlobe shapes by race [29]. The prevalence of ELC in Japanese adults is low compared with those in Europe and America [20]. The subjects in the present study were limited to Asian Chinese, which limit the generalizability of the findings to other populations. In view of the possible cultural differences on the presentation of ELC, further studies on earlobe creases should therefore take into consideration the impact of age, race, and earlobe shape on ELC prevalence. 

Although retrospectively examining the auricles from the time of birth of these subjects is not possible, we believe that the crease is not present at birth and develops later in life. However, whether the crease is a genetic predisposition that takes years to appear or whether it is a result of localized vascular disease and skin atrophy remains to be determined [21]. Shoenfeld et al. [33] speculated that a diminished blood supply to the earlobe might contribute to the elastic fiber tears that result in creases and folding. Kirkham et al. [34] concluded that a strong association exists between earlobe creases and an increased risk of a cardiovascular cause of death in their study involving 303 coroner necropsies. Their finding agrees with a prospective study, which suggested that a diagonal ELC is associated with all causes of cardiac morbidity and mortality [5]. 

We also found that the degree of stenosis of RCA was significantly associated with the presence of ELC and the number of major epicardial arteries involved was also associated with the presence of ELC and of edema around the “Heart” zone. Such findings corroborate with a previous study conducted on 376 postmortem cases by Patel et al., who also reported a strong association between severe coronary atherosclerosis and increasing ELC grading [10]. Elliott and Powell further confirmed that such relationship appeared in a graded fashion on patients admitted to the hospital with suspected coronary disease [35]. The result aligned with the findings of Pasternac and Sami that the ear-crease sign was significantly more common among patients with grade 3 (narrowing of the lumen of three main vessels greater than 50.0%), which was identified via coronary arteriography [31]. Tranchesi et al. also found that the number of vessel blockage was associated with the prevalence of ELCs among the cases in their study [6]. Furthermore, an association between increased carotid intima-media thickness, a surrogate marker for atherosclerotic disease, and diagonal ELC was noted in healthy subjects [36]. 

More participants in the CHD+ve group had hair growth on ears than those in the CHD−ve group. Such finding corroborate with the observations reported by Verma et al. that excessive growth of hair in the meatus externa has a correlation with CHD [37]. 

### 8.2. Electrical Skin Resistance Measurement

The “Heart” zone of CHD+ve cases showed significantly higher skin conductance on both ears (i.e., less electrical skin resistance) than the control group. The electrical resistance in the corresponding auricular points decreases when a disease or disorder is present in the body, and areas with low electrical resistance than that of the standard are considered either positively or highly conductive electrical resistance points [1]. Several possible reasons may explain this phenomenon. From a physiological perspective, the changes in electrical resistance on the auricular points can be the result of a change in the electrical resistance on the underlying cell membranes [38]. The electrical resistance on the cell membranes on a particular bodily system would be lower when it is not functioning well. The signal is then transmitted to the central nervous system through the meridians. The nerve cells stimulated in the central nervous system would send signals to the corresponding auricular points and consequently change its electrical resistance [38]. In a study conducted by Oleson et al. [14] on cases with musculoskeletal pain, they hypothesized that a somatotopic organization of the body is represented on the human auricle, and such alterations in skin conductivity at painful areas of the body have been attributed to the regional hyperactivity of the sympathetic nervous system. Moreover, the activation of sudomotor sympathetic nerves may cause a change in skin moisture level and result in a decrease in electrical resistance [39]. In the present study, a significant change was observed in the conductivity of the “Heart” zone in chronic CHD+ve cases, but not in acute cases. The change in electrical conductivity of acupoints possibly needs some time to develop and is present when the disease progresses. 

### 8.3. Tenderness Testing

The participants in the CHD+ve group experienced significant tenderness in the “Heart” region than those in the CHD−ve group in both acute and chronic cases as detected by the pressure algometer (force gauge). The degree of tenderness is usually related to the severity of the condition; the more sensitive the point, the more severe the disorder [3, 40]. In an animal experiment, Oleson [41] found that the skin acupuncture points on dog bodies showed significantly higher concentrations of substance P than that of the control skin points. Substance P is a spinal neurotransmitter found in nociceptive, afferent C-fibers, which helps in pain transmission and triggers the subcutaneous release of histamine. Thus, the sensory neurons become hypersensitive. As a result, an increase in substance P concentration would decrease the pain threshold and make the auricular points tender when touched [41]. Among the three examination methods adopted in the current study, tenderness testing of the “Heart” zone has the highest sensitivity (92.0%) and the highest positive predictive value (78.6%) for the prediction of CHD status. 

Based on the multivariate logistic regression analyses, the presence of ELC on the left side of the ear as well as the tenderness and conductivity in the “Heart” region of the left ear were notably associated with the CHD status after the adjustment of the smoking status and MAP readings. The occurrence rate of ELC on the left side of the ear was found to be slightly higher than that on the right ear. This phenomenon may possibly be explained by the reflex pathway of the “Heart”, which is more likely to occur on the left side of the ear. This trend is similar to the pictograph of foot reflexology, which shows that the “Heart” is located on the left foot and not on the right foot. Moreover, the said phenomenon may be associated with the fact that the LAD artery in these subjects was most commonly involved in blockage (75.0%).

## 9. Implications of Findings

The results of this study can advance our knowledge in understanding the association between specific auricular reflective signs and CHD status. Such information will be a valuable background data to support future studies for screening vulnerable populations with CHD risk, such as those with family history, hypertension, hyperlipidemia, sedentary lifestyle, and obesity. Auricular diagnosis has a pre-diagnostic value and is important in the secondary level of prevention. Prognosis may be improved by early preventive measures if CHD status can be identified at an earlier stage. 

The examination of the auricles may be integrated into the routine clinical examination of a patient to increase the predictive accuracy of the coronary risk profile for earlier introduction of preventive measures, such as control of hypertension and smoking, before the disease advances. This process may help detect CHD in its early stages and shed some light on the early development of coronary atherosclerosis. ELC may be a useful indicator for the presence of CHD in the preoperative assessment of patients, particularly in emergency situation, where little or no history and investigations are available. The use of ELC may improve the clinician's ability to estimate the probability of CHD in patients before they undergo diagnostic testing and to predict the operative risk [7, 20]. Given that the ear is a valuable tool in revealing constitutional predispositions, the use of auricular diagnosis, if found effective, can be used as a complementary approach that is simple, effective, and inexpensive for identifying patients with coronary risks.

## 10. Limitations of Study

Although the association between ELC and CHD is suggested in this study, the mechanisms leading to the concurrent development of ELC and CHD and the time of onset remains uncertain. A prospective cohort study could be conducted in the future to follow-up the newly diagnosed CHD+ve cases and to identify changes in the auricular signals that may have appeared during disease progression. This study was conducted on a relatively small sample. Therefore, further investigations must be performed with a larger sample to validate the use of auricular signs as predictors that assist in the diagnosis of CHD. Due to resource implications, the CHD−ve status can only be verified by self-report of the participants in the control group who do not have any previous medical history of CHD or any experience of having cardiac-related symptoms, for example, palpitation, chest pain, and dyspnoea. The prevalence of ELC may be governed by ethnical differences; thus, more work is required before these results can be extrapolated to other ethnic groups.

## 11. Conclusion

The predictive value of auricular signals on CHD, which was detected by visual inspection, electrical skin resistance measurement, and tenderness testing, was observed in the Chinese population in this study. Such findings can advance our knowledge on the integrated approach that combines Chinese and Western models of care for diagnosing patients with underlying CHD. Further studies should be conducted to ascertain the diagnostic value of AT on CHD. 

## Figures and Tables

**Table 1:** The demographic and clinical risk factors of the participants.

	CHD+ve (*n* = 50)	CHD−ve (*n* = 50)	Test statistics McNemar (unless indicated)
Age	65.24 (14.51)	65.40 (13.86)	NS
Gender			
1: male	27	27	NS
2: female	23	23	
Employment status			
1: full time, part time	17	15	NS
2: retired/housewife/unemployed	33	35	
BMI	25.13 (5.02)	24.42 (4.19)	NS
Comorbid illness			
0: no	29	30	NS
1: yes	21	20	
Smoking status			
1: never	28	39	0.019
2: ex-smoker, current smoker	22	11	
Family history on MI			
1: no	37	42	NS
2: yes	13	8	

Mean arterial pressure (MAP): diastolic BP + (systolic − diastolic BP)/3	89.32 (9.61)	93.25 (9.27)	(Paired *t*-test) *t* = −2.271, df = 49, *P* = 0.028

NS: nonsignificant.

**Table 2:** Final multivariate logistic regression analysis of coronary heart disease status.

Variable	Adjusted odds ratios	95% CI	*P* value of Wald statistic
Ear lobe crease (Left) (%)	1.02	1.00–1.04	0.047*
Conductivity of “Heart” region(Left)^*⋇*^			
Present	5.67	1.20–26.69	0.002*
Absent	1.0		
Tenderness of “Heart” region(Left)^*⋇*^			
Present	49.99	11.81–211.58	<0.0001*
Absent	1.0		
Mean arterial pressure	0.94	0.87–1.01	0.087
Smoking status			
Ex-smoker, current smoker	3.48	0.76–15.85	0.108
Never	1.0		

*Significant *P* value.

CI: Confidence interval.

^*⋇*^comparedwith reference point “Shenmen.”

## Abbreviations

CHD: Coronary heart disease
ELC: Ear lobe crease
BMI: Body mass index
PPV: Positive predictive value
NPV: Negative predictive value
CI: Confidence interval
SD: Standard deviation
MAP: Mean arterial pressure
ROC: Receiver operator characteristics
AUC: Area under curve
OR: Odds ratio
LAD: Left anterior descending
RCA: Right coronary artery
LCX: Left circumflex
PDA: Posterior descending artery
PLB: Posterior lateral branch.
